# Senescence and Cancer: Role of Nitric Oxide (NO) in SASP

**DOI:** 10.3390/cancers12051145

**Published:** 2020-05-02

**Authors:** Nesrine Mabrouk, Silvia Ghione, Véronique Laurens, Stéphanie Plenchette, Ali Bettaieb, Catherine Paul

**Affiliations:** 1 Laboratory of Immunology and Immunotherapy of Cancers, EPHE, PSL Research University, 75000 Paris, France; nesrine_mabrouk16@yahoo.fr (N.M.); silvi.ghio50@gmail.com (S.G.); veronique.laurens-calin01@u-bourgogne.fr (V.L.); Stephanie.Plenchette-Colas@u-bourgogne.fr (S.P.); ali.bettaieb@u-bourgogne.fr (A.B.); 2 Laboratory of Immunology and Immunotherapy of Cancers (LIIC), EA7269, University of Burgundy Franche-Comté, 21000 Dijon, France

**Keywords:** SASP, nitric oxide, cancer treatments

## Abstract

Cellular senescence is a cell state involved in both physiological and pathological processes such as age-related diseases and cancer. While the mechanism of senescence is now well known, its role in tumorigenesis still remains very controversial. The positive and negative effects of senescence on tumorigenesis depend largely on the diversity of the senescent phenotypes and, more precisely, on the senescence-associated secretory phenotype (SASP). In this review, we discuss the modulatory effect of nitric oxide (NO) in SASP and the possible benefits of the use of NO donors or iNOS inducers in combination with senotherapy in cancer treatment.

## 1. Senescence and Cancer

Senescence is an important biological mechanism that plays a crucial role in many physiological processes, such as development and wound healing, and also in various age-related pathologies including cancer [1,2,3]. Indeed, there is a strong relationship between aging and cancer. In both cases, the mechanisms involve an accumulation of cellular damage over time. However, other processes seem to be in opposition, such as hyperproliferation and the increase in cell survival in cancer or the decreased function and fitness in aging [4]. However, many hallmarks of aging are found in cancer, and senescence is one of them [5]. Senescence was first described in 1961 by Hayflick and Moorhead after observing that human diploid cells have a replicative limit in culture [6]. This process, named replicative senescence, is the first form of senescence described and involves the shortening of telomeres [7]. The loss of telomeres is recognized as DNA damage and triggers a DNA-damage response (DDR), involving DNA damage kinases such as ataxia-telangiectasia-mutated protein (ATM), ataxia-telangiectasia and Rad3-related protein (ATR) and checkpoint kinases 1 and 2 (CHK1 and CHK2), which in turn activate cell cycle proteins such as p53 [8]. DDR is also induced by other external damaging factors such as ionizing radiation and chemotherapeutic drugs [9]. Other forms of non-telomeric senescence, called premature senescence, have been described such as stress-induced senescence, which involves reactive oxygen species (ROS), and oncogene-induced senescence (OIS). OIS was firstly described by Serrano et al. (1997) in a model of human fibroblasts expressing an oncogenic form of RAS^G12V^ [10]. Since then, more than 50 oncogenes have been described to induce senescence once activated [11]. In most OIS cases, the senescent phenotype is invariably preceded by hyperproliferation, aberrant replication and activation of the DDR [12,13]. PICS, PTEN-loss induced cellular senescence [14] is also a form of premature senescence, occurring independently of DDR or hyper-replication [15]. The loss of two other tumor suppressors, neurofibromin 1 (NF1) and the von Hippel-Lindau factor, has also been described to induce senescence in the same manner as for PICS [9].

The senescent phenotype is often characterized by the modulation of different signaling pathways and a series of cellular events such as the activation of the DDR; cell cycle arrest via the activation of the p53/p21^CIP1^ and p16^INK4A^/Retinoblastoma (Rb) pathways; the induction of a senescence-associated secretory phenotype (SASP); resistance against apoptosis; the induction of endoplasmic reticulum stress; and the modulation of metabolism [16]. Since all these biological aspects are not specific to the senescence process, multiple markers should be considered to accurately define the senescent phenotype. The most commonly used assay is based on the histochemical detection of Senescence-Associated beta-galactosidase (SA-β-gal) [17]. Other canonical senescence markers include senescence inducers such as CDK inhibitors (p16, p21, p15 and p27) and the phosphorylated forms of p53 and Rb, which are proteins related to senescence-associated heterochromatin foci (SAHF) and the expression of extracellular factors associated with SASP [9]. In vitro morphological changes are also a common feature of senescence. Indeed, senescent cells are often characterized by structural cell changes such as enlarged morphology, the modification of plasma membrane composition, the accumulation of lysosomes and mitochondria, and nuclear changes [16].

Cellular senescence was primarily described as a tumor suppressor mechanism [14,18,19,20]. Indeed, first of all, senescence can inhibit the growth of cancer cells and then induce SASP, which in turn induces the recruitment of immune cells. However, the aging of tumor microenvironment and genetic and epigenetic mutations induced by tumor progression cooperate to bypass senescence [4]. Moreover, the long-term implications of senescent cells—and more particularly, the paracrine effect of SASP—potentially favor tumor development [21,22,23,24]. Recently, in a very comprehensive review, Faget et al. (2019) highlighted the existence of different SASPs in immunosupportive or immunosuppressive scenarios [25]. To counteract the pro-tumorigenic effects of SASP, new therapies have also been developed to dampen SASP’s proinflammatory effects (senomorphic) or to specifically clear senescent cells (senolytics) [26]. These senotherapies selectively kill senescent cells or stimulate their elimination by the immune system [27]. These approaches include chemical inhibitors of the nuclear factor-kappa B (NF-κB), Janus kinase (JAK) and mammalian target of rapamycin (mTOR) pathways and free radical scavengers [28,29,30].

Nitric oxide (NO) might be included in these therapies because a lot of pathways are regulated by post-translational modifications—such as cysteine S-nitrosylation (Cys-NO), tyrosine nitration (Tyr-NO) or even metal nitrosylation [31]—and because NO is also strongly implicated in immunomodulation [32]. Here, we describe the involvement of NO in senescence via (1) the modulation of SASP pathways, (2) the regulation of apoptosis via death receptor pathway modulation, (3) immunomodulation and (4) the modulation of the senolytic effects. Altogether, these data pave the way towards the development of new therapeutic strategies based on NO.

## 2. SASP in Cancer: A Double-Edged Sword

It is now well established that senescent cells are able to secrete a pool of molecules that define the SASP. This condition mediates the paracrine activity of senescent cells, which results in tissue microenvironment modulation [25]. Many SASP components are actually identified, such as growth factors, cytokines, chemokines, proteases and extracellular matrix (ECM) components [26,33]. SASP soluble factors include, among others, interleukin 6 (IL-6), IL1α, IL-10, IL-13, IL-15, chemokines such as CC motif ligand 2 (CCL2), CCL5, CCL8 and C-X-C motif 1 (CXCL1), CXCL2, CXCL5, CXCL12, granulocytes macrophage colony stimulating factor (GM-CSF), macrophage colony stimulating factor 1 (CSF1), and cytokines such as interferon γ (IFNγ), tumor necrosis factor α (TNFα), insulin-like growth factors (IGFs) and transforming growth factor β (TGFβ) [33,34]. In addition to soluble factors, senescent cells can secrete proteases, such as matrix metalloproteinases (MMPs) and serine proteases, and regulators of plasminogen activation pathways, which induce ECM modifications. In fact, it is well established that ECM changes (e.g., rigidity loss) increase the metastatic properties of cancer cells and provide optimal conditions for migration. These changes in the ECM are associated with poor prognosis in cancer patients [35]. For this reason, these molecules are involved in carcinogenesis and cell migration. MMP family members involved in SASP are stromelysin-1 and -2 (also known as MMP-3 and -10) and collagenase 1 (MMP-1), while plasminogen activators include urokinase (uPA), tissue-type plasminogen activators (tPA), uPA receptor (uPAR) and inhibitors of these serine proteases (PAI-1 and 2) [24,36]. The SASP factors are summarized in Table 1.

The SASP molecules can modulate stromal and immune cells, with a different role in cancer development. In fact, SASP is considered as a double-edged sword because it can lead to either anti-tumorigenic or a pro-tumorigenic effects in a context-dependent manner. Indeed, several SASP factors induce the clearance of senescent pre-neoplastic cells, preserving tissue homeostasis. During the early stage of senescence transformation, these cells are recognized and eliminated by the immune system recruited by SASP, to prevent the malignant transformation [37]. For example, a high level of TGFβ secreted by macrophages in the tumor microenvironment maintains cellular senescence and decreases tumor growth in aggressive B cell lymphoma [38]. Alessio et al. (2019) recently showed that the induction of acute SASP (A-SASP) in mesenchymal stem cells (MSCs) decreases immortalized prostate cancer cell PNT2 proliferation in vitro [39].

Nevertheless, long-term exposure to SASP factors or the aging of the tumor microenvironment increases inflammation and tumor progression [4,25]. Therefore, senescent cells can also contribute to tumorigenesis via SASP [40]. The accumulation of cytoplasmic DNA in senescent cells is able to activate the GMP-AMP synthase (cGAS)-stimulator of interferon genes (STING) pathway, causing chronic inflammation. cGAS binds to dsDNA, and this condition causes conformational changes in its catalytic center, inducing the catalysis of cyclic GMP-AMP (cGAMP). This second messenger stimulates STING, an endoplasmic reticulum-located protein, which induces the activation and nuclear translocation of interferon-regulatory factor 3 (IRF3) and NF-κB. This causes the expression of, for example, type I IFN, a crucial player in inflammation [41]. STING activation is associated with inflammatory diseases, and the link between inflammation and cancer is well recognized [42]. For example, several studies reported the importance of the STING signaling pathway in 7,12dimethylbenz[α]anthracene (DMBA)-induced carcinogenesis. This compound can drive the development of skin tumors by DNA damage, which induces STING-dependent pro-inflammatory cytokine production [43,44]. Furthermore, a recent study has shown that STING-deficient mice are resistant to DMBA-induced skin tumors [45]. Another study showed that STING activation induces obesity-associated hepatocellular carcinoma (HCC) in mice [46]. The over-activation of the cGAS-STING signaling pathway was also described in lung cancer [47].

Although chemotherapy is beneficial to treat cancer, these compounds can promote SASP secretion via increased DNA damage, leading to inflammation and subsequent cancer progression [48]. It is actually known that several anti-cancer drugs, such as docetaxel, bleomycin, cyclophosphamide, doxorubicin, vincristine, etoposide, 5-fluorouracil (5-FU), cisplatin and also ionizing radiation can mediate tumor-induced senescence [34,49]. This mechanism is principally mediated by DNA damage and inflammation in the tumor microenvironment. In this context, Tato-Costa et al. (2016) showed that the SASP induced by 5-fluorouracil (5-FU) and doxorubicin causes epithelial-to-mesenchymal transition (EMT) in vitro and in clinical samples from patients with rectal cancer [49]. Another important factor included in SASP is NO [50]. Senescent cells are able to secrete NO, via nitric oxide synthase (NOS). In the SASP microenvironment, fibroblasts are major NO producers, enhancing cancer cell proliferation. It is well known that NO is involved in monocyte differentiation, and these cells, under certain conditions, can induce cancer progression [51,52]. Moreover, NO is also involved in the modulation of a large number of signaling pathways [53].

## 3. Role of NO in the Induction of the SASP

According to previous reports, several mechanisms are involved in the regulation of SASP factors. Considering the large number of signaling molecules involved in SASP secretion, the present review is focused on the most crucial ones. The transcription factor NF-κB plays an important role in SASP. It can be activated by several SASP inducers such as toll like receptor 2 (TLR2), TNFα, reactive oxygen species (ROS) and genotoxic agents [54,55]. The activation of NF-κB involves IκB phosphorylation by IκB kinase (IKK), which leads to its degradation by the proteasome, enabling the active NF-κB transcription factor (p65/p50) to translocate into the nucleus and thereby induce the expression of target genes such as those which code for IL-6, IL-8 and CXCL1 [56,57,58]. Other senescence activators such as CXCR2 ligands IL-8 and GRO/Gro1 are also upregulated in cells undergoing senescence following the activation of mitogen-activated kinases [59]. Recently, Loo et al. (2017) reported that the expression of cyclooxygenase 2 (COX-2), a rate limiting enzyme involved in prostaglandin biosynthesis, is increased in senescent hepatic stellate cells and triggers the overproduction of the SASP prostaglandin E2 (PGE2) via PGE2 receptor EP4, limiting anti-tumor immunity in obesity-associated HCC [60].

The identification of signals that can promote senescence in a given tumor type may provide new therapeutic targets for cancer. Several drugs (see below) modulate some of these signals, including NO.

NO, a highly reactive free radical, has pleotropic functions in multiple biological processes, such as neurotransmission, vasodilatation and macrophage-mediated immunity. NO is synthetized from L-arginine by NOS in the presence of oxygen. Endothelial NOS (eNOS) and neuronal NOS (nNOS), two NOSs whose activities are dependent on calcium levels, produce low amounts of NO, while the third NOS is inducible (iNOS) and independent of calcium levels. iNOS is activated by IL-1β, Toll Like 4 receptor agonists (TLR4), interferon γ (IFNγ) and oxidative stress and produces higher amounts of NO [61]. As a very unstable molecule, NO reacts mainly with the superoxide anion to generate peroxynitrite, a compound with high oxidative potential [62].

NO is also released from pharmacological agents. There are direct NO donors—such as sodium nitroprusside, molsidomine or diethylamino-NONOate—and donors that require metabolism such as the classic nitrovasodilators and organic nitrate and nitrite esters—including nitroglycerin (also called glyceryl trinitrate or GTN), isosorbide dinitrate, isosorbide 5-mononitrate and nicorandil—that have been used in the treatment of cardiovascular diseases [63,64,65].

In cancer, NO plays a role in promoting as well as inhibiting tumors [62,66,67]. This dichotomy seems to be related to its concentration, its location and its targets [68]. A few clinical studies have shown that NO donors could mediate anti-tumor activities used alone or in combination with standard therapies [69,70,71]. Indeed, in the first phase II clinical trial, the use of nitroglycerin combined with vinorelbine and cisplatin improved the overall response and time to disease progression in patients with non-small-cell lung cancer (NSCLC) [69]. In a second phase II study, the addition of nitroglycerin to cisplatin and vinorelbine and concurrent radiotherapy in patients with advanced NSCLC had an acceptable toxicity profile and the possibility of adding nitroglycerin to chemotherapy and radiotherapy was supported [70]. In another phase II study conducted in patients with prostate cancer, nitroglycerin increased the prostate-specific antigen (PSA) doubling time and the safety of the drug was confirmed [71].

**Figure 1:**
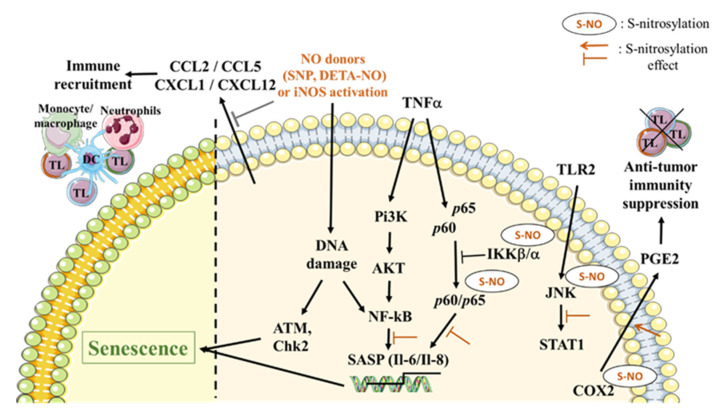
Impact of nitric oxide (NO) in senescence-mediated signaling pathways. NO plays important roles in different senescence-associated secretory phenotype (SASP) signaling pathways. Exogenous and endogenous NO can cause DNA damage that can lead to protein kinase ataxia-telangiectasia-mutated protein (ATM) and Chk2 activation, or NF-κB activation and interleukin 6 (IL-6)/IL-8 gene transcription. All these changes can induce the initiation of cellular senescence programs. NO is also able to induce direct modifications in NF-κB, either by S-nitrosylating both p60/p65 NF-κB subunits and leading to SASP gene transcription inhibition, or by S-nitrosylating the NF-κB kinase inhibitor subunit α (IKKα), which results in NF-κB inhibition. S-nitrosylation can also affect the c-Jun N-terminal kinase (JNK) signaling pathway by leading to the inhibition of its activation. Moreover, cyclooxygenase 2 (COX2), a prostaglandin E2 (PGE2) secretion inducer, is also an NO target. The S-nitrosylation of COX2 stimulates its activity and induces anti-tumor immunity suppression. Another group of SASP factors altered by NO are chemokines such as CCL2, CCL5, CXCL1 and CXCL12, which can transcriptionally and translationally be decreased in response to NO donors, thus reducing immune cell recruitment.

NO-induced survival or cell death seems to be related to its biochemical action on proteins by causing post-translational modifications [31]. S-nitrosylation, the transfer of NO to a free-SH group of a specific cysteine residue, is now considered to be essential for regulating the function of many proteins and signaling pathways including those involved in cancer regulation [72]. It modulates protein structure, function, expression, location or interaction with other protein partners [73]. The ambivalent nature of NO in cancer is dictated by the impact of S-nitrosylation on proteins involved in signaling pathways that trigger both survival and cell death. Thus, NO and its derivatives, such as peroxynitrites, are able to cause direct or indirect DNA damage. Direct damage includes DNA base deamination, adduct formation and single strand breaks in the DNA. Indirect damage is due to the interactions of NO reactive species with other molecules such as amines, thiols or lipids [74]. Furthermore, NO and its derivatives modulate SASP factors. Recently, the NO donors sodium nitroprusside dihydrate (SNP) and diethylenetriamine/nitric oxide adduct (DETA/NO) have been reported to cause DNA double-strand breaks (DSBs), initiating cellular senescence programs in numerous cell lines of different origins (cervical and lung cancers, fibroblastic cell lines). This effect is associated with the activation of the protein kinase ATM, an upstream activator of the DDR; the activation of NF-κB; and an increase in SASP factors like IL-6 and IL-8 [75]. NO can also regulate other pathways involved in the secretion of SASP factors such as NF-κB and the mitogen activated protein kinase c-Jun N-terminal kinase (JNK) pathways [76]. Indeed, Reynaert et al. (2004) have reported that NO triggers the S-nitrosylation of the inhibitor of NF-κB kinase subunit β (IKKβ, a regulator of the classical NF-κB pathway activation) at cysteine residue 179 and consequently results in NF-κB inhibition [77]. In addition, NO can S-nitrosylate both the p50 and p65 NF-κB subunits at cysteine residues 62 and 38, respectively, reducing their DNA binding and inhibiting target gene transcription [78,79]. Moreover, the tyrosine nitration of IκBα at tyrosine 181 by endogenous NO promotes NF-κB signaling through the dissociation of IκBα from NF-κB [80]. The endogenous production of NO or NO donors such S-nitro-N-acetyl-penicillamine (SNAP) can suppress JNK activation via S-nitrosylation at cysteine 116 [81]. However, the relationship of NO-induced senescence with its ability to target NF-κB and JNK has never been reported so far (Figure 1).

NO also targets some cytokines that belong to SASP factors. For instance, the NO-aspirin derivative NCX-4016 (100 mM) inhibits the release of numerous cytokines (IL-1β, IL-18, IL-8, IL-12, IFNγ and TNFα) in monocytes isolated from human PBMCs of healthy donors after challenge with the bacterial endotoxin, lipopolysaccharides (LPS) (1 μg/mL). This effect is due to the inhibition of IL-1β Converting Enzyme, also named caspase-1, required for intracellular processing/maturation of IL-1β and IL-18 [82]. These data may suggest that in the context of senescence, the NO-mediated inhibition of these pro-inflammatory cytokines could alleviate cancer development, invasion, and metastasis [83]. Furthermore, NO also targets another SASP factor, the signal transducer and activator of transcription 3 (STAT3), an important target in cancer therapy and a key kinase involved in the IL-6 signaling pathway. Indeed, STAT3 is regulated by the S-nitrosylation of the cysteine residue at position 259, which inhibits STAT3 phosphorylation, its downstream activation and affects IL-6-mediated cell proliferation [84]. As mentioned before, COX2 expression is significantly increased in senescent cells. The overexpression of PGE2 (the major COX-2 product), which functions as a key SASP factor in the tumor microenvironment, suppresses the anti-tumor immunity and progression [60]. A previous study has established that NO can modulate COX2 activity. Indeed, Kim et al. (2005) showed that iNOS specifically binds to the enzyme COX2, induces its S-nitrosylation and enhances its catalytic activity [85]. NO can also modulate immune cell recruitment through its action on chemokines, another group of SASP factors. Indeed, Giustizieri et al. (2002) have reported that the NO donor, S-nitrosoglutathione (GSNO), diminished, in a dose-dependent manner, both the mRNA and protein levels of CCL5 (RANTES) and CXCL1 (GROα) in keratinocytes cultured from healthy or psoriatic patients [86]. These results were confirmed in another cellular model by Kashiwagi et al. (2002,) which showed that CCL5 and CXCL1 are up-regulated in renal cortex of rats chronically treated with an NO synthase inhibitor, and induced the recruitment of monocytes/macrophages [87] (Figure 1).

## 4. NO Involvement in Expression/Activation of Death Receptors and Death Ligands 

The tumor necrosis factor (TNF) ligand family members TNFα, Fas ligand (FasL) and Tumor-necrosis-factor related apoptosis ligand (TRAIL) are major immunoregulatory cytokines of the tumor microenvironment, also found to be important SASP components (Table 1) [24]. These cytokines exert paradoxical functions, either by sustaining tumor growth and chemoresistance or by killing tumor cells, in the tumor microenvironment in a context-dependent manner [88]. One anti-tumor strategy would consist of switching the roles of TNFα, FasL and TRAIL from their pro-apoptotic functions to favor tumor cell death. A growing number of studies indicate that NO can regulate the signaling pathways driven by TNFα, FasL and TRAIL at many different levels [89,90]. Several studies have indicated that NO may exert dual effects in cancer, that can lead, on one hand, to enhanced tumor growth and progression (genotoxicity, apoptosis resistance, angiogenesis, invasion and metastasis) and on the other hand, to tumoricidal effects (cytostatic and/or cytotoxic effects on tumor cells) [91,92]. The biological impact of NO relies on various aspects (e.g., the NO concentration, tumor redox microenvironment and duration of NO exposure). Thus, NO donor-based therapies are currently under investigation to further sustain the tumoricidal effect of NO.

Several reports have demonstrated the role of NO-mediated sensitization of cancer cells to apoptosis in many ways [88]. Thus, NO-based therapies could represent a new potential strategy to reduce the threshold of cancer cell resistance. Various NO donors are under investigation to understand the molecular mechanisms that underly their modes of action. It is becoming even more evident that the post-translational modifications of selective proteins by NO exert an important regulatory control, either positive or negative, over various signaling pathways engaged by TNF ligands and their receptors [88,93]. The cellular response at least relies on the relative threshold of NO production (either endogenous or from NO-releasing drugs), cellular context and severity of oxidative stress. If the biological outcome of the NO-induced S-nitrosylation of Fas [94], DR4 [95] and TNFR1 [96] results in cell death induction, the molecular mechanisms that control this process have not been fully unraveled yet. Importantly, the NO donor GTN can mediate the S-nitrosylation of Fas at cysteine 304, which consequently leads to its aggregation into lipid rafts to stimulate downstream signaling and cancer cell death [94]. In agreement, the S-nitrosylation of the transcriptional repressor Ying Yang 1 (YY1) inhibits its DNA-binding capacity to the silencer region of Fas promoter and then up-regulates *FAS* gene expression and consequently sensitizes cells to apoptosis [97,98].

The S-nitrosylation of TNFR1 by the NO donor NONOate has also been described in hepatoma cells; however, the exact targeted cysteine residue is unknown [96]. As previously described, the classical NF-κB pathway activated by the TNFα/TNFR1 system is controlled by the S-nitrosylation and nitration of specific target proteins at different levels of the molecular pathway. More recently, the S-nitrosylation of cIAP1 (a positive regulator of the NF-κB signaling pathway), induced by the NO donor GTN (particularly at cysteine 571), appeared as a critical cornerstone for switching the cancer cell fate from TNFα/TNFR1-mediated cell survival (through the activation of the classical NF-κB cascade) to TNFα/TNFR1-mediated cell death [90].

To date, the regulatory role of NO in the TRAIL/TRAIL receptors (TRAILR) system is less documented. Only DR4 was reported to undergo S-nitrosylation and, furthermore, by a specific NO donor (Nitrosylcobalamin), particularly at cysteine 336, and consequently foster cancer cell apoptosis [95]. Accordingly, NO disrupts the transcriptional repressor activity of YY1 not only on *FAS* but also on *DR5*, up-regulates its expression and sensitizes cancer cells to TRAIL-induced apoptosis [99].

Several crucial factors of apoptosis involved in the TNF signaling pathways, such as caspases, Bcl-2 family proteins or FLIP, can undergo post-translational modifications by NO that would impact cell fate [88,93]. Although SASP components include NO, the S-nitrosylation of Fas, DR4 and TNFR1 was demonstrated exclusively via the NO released by NO donors.

Interestingly, beside their dichotomous responses in cancer, TNF ligands, particularly TNFα and FasL, can lead to the process of senescence. Indeed, cancer cells’ senescent phenotype can arise in response to TNFα/TNF receptor 1 (TNFR1) through the activation of the p16^INK4A^/Rb pathway [100]. The senescent phenotype can also occur in response to FasL/Fas in a context-dependent manner, particularly in microsatellite instability-high type colon tumors. Mechanistically, Fas-induced senescence was caused by a chronic DNA damage response via caspase-activated DNAse resulting in p53 activation and p21 expression [101].

Whether the SASP could modulate the TNF ligand systems via NO, either in an autocrine or paracrine manner, remains to be demonstrated.

## 5. Role of NO in the SASP-Immunomodulatory Effect

Because senescent cells remain viable and exhibit the SASP phenotype with a wide spectrum of diverse physiological functions, their existence in the tumor mass can have an ambivalent impact, from tumor regression to promotion. SASP cytokines act on the recruitment and activation states of immune cells. They can cause the tumor infiltration of immunosuppressive cells like macrophages and myeloid-derived suppressor cells (MDSCs), thus promoting tumor growth. Conversely, they can also induce the tumor infiltration of natural killer cells (NK) and effector T lymphocytes and thus have anti-tumor properties [102].

In recent years, NO has emerged as an important immunomodulatory agent in the tumor microenvironment [32,103]. In situ, different sources of endogenous NO can be considered. Indeed, NO can be produced by various types of cell expressing iNOS and eNOS. Moreover, NO, as well as ROS, can also derive from senescent cells as non-macromolecular components of the SASP [24] (Table 1). It has been demonstrated that the tumor microenvironment has all the conditions for iNOS expression and NO production, which is important for the maintenance and progression of an aggressive tumor phenotype in breast cancer [104]. iNOS has an immunosuppressive role within the tumor microenvironment via its actions on MDSCs as well as via the loss of the effector function of cytotoxic T lymphocytes (CTLs). Furthermore, two studies have shown that two NO-releasing drugs—NO-aspirin and NO-aspirin derivative (AT38)—induced, in numerous types of cancer, a feedback inhibition of iNOS in MDSCs [105,106]. Such effects result in the decreased MDSC-induced nitration of T-cell receptors, a massive infiltration of the tumor by T-cells and an enhanced efficacy of DNA cancer vaccination [105,106]. Indeed, NO from iNOS-expressing tumor cells disturbs the polarization and directional secretion of cytotoxic granules in the immune synapse of tumor infiltrating lymphocytes (TILs) [107]. Nitration by iNOS-derived NO is also involved in the polarization of tumor-associated macrophages. NO leads to the suppression of the M1 macrophage signature gene activation and induces a pro-tumorigenic environment [108].

Among the SASP chemokine data for nitration, CCL2 and CXCL12 have been reported. These chemokines interact with the G-protein coupled receptors CCR2 and CXCR4, respectively, and bind to glycosaminoglycans (GAGs) present on the surface of endothelial cells and in the extracellular matrix. CCL2, also known as monocyte chemoattractant protein-1 (MCP-1), greatly contributes to the recruitment of monocytes, memory T cells and dendritic cells into sites of inflammation and tumors. It has been shown that peroxynitrite-treated CCL2 lost its ability to recruit CD8^+^ T cells, but the recruitment of myeloid-derived suppressor cells was unaltered [106]. Nitrated CCL2 has a reduced affinity to its receptor CCR2, which may explain its failure to induce the chemotaxis of CD8^+^ T cells expressing low levels of the CCR2 but the retention of its ability to induce the migration of myeloid cells expressing high levels of CCR2 [109]. CXCL12, also known as stroma cell-derived factor-1 (SDF-1), represents the single natural ligand for the chemokine receptor CXCR4 and induces the activation and migration of most leucocytes. The nitration of tyrosine 7 in CXCL12 hampers the chemokine’s ability to induce lymphocyte chemotaxis (Figure 1). This nitration of CXCL12 does not affect its ability to bind to the CXCR4 receptor but does impair its ability to signal through this receptor. If nitrated CXCL12 binds to GAGs with a similar affinity as wild type CXCL12, nitrated CCL2 has a reduced ability to bind GAGs compared to wild type CCL2, and therefore could limit further leukocyte chemotaxis. When nitration reduces receptor activation capacity without affecting receptor affinity, this influences the receptor’s signaling in situations where many chemokines can bind the same receptor [110].

NO from MDSCs has been described to suppress T lymphocyte proliferation via the suppression of STAT5 phosphorylation [111]. However, as we have underlined above, the immunosuppressive effect of MDSCs can be constrained by NO-releasing drugs [105,106]. NO is also involved in STAT3 phosphorylation through src homology protein tyrosine phosphatase 2 (SHP2) nitration, known as a negative regulator of STAT3 phosphorylation [112]. This study was conducted in a mouse model of ulcerative colitis, and the impact of NO on STAT3 phosphorylation was investigated in LPS-activated macrophages but not in a tumor context or in T lymphocytes in whose activation STAT3 plays a key role.

SASP is also involved in immune resistance mechanisms, since high levels of IFNγ drive the expression of the programmed death ligand 1 (PD-L1), one of the targets of immune checkpoint inhibitor therapy (ICT). In tumors, high expression of the transcription factor YY1 modulates PD-L1 expression. Treatment with NO donors results in the inhibition of PD-L1 expression via the S-nitrosylation of YY1 [113]. By a nitroproteomic approach, authors uncover a potential mechanism for the ICT where a key protein for T cell activation is nitrated and inactivated by MDSCs. Indeed, lymphocyte-specific protein tyrosine kinase (LCK), an initiating tyrosine kinase in the T cell receptor signaling cascade, is nitrated at tyrosine 394 by MDSCs [114]. After ICT, high expression of iNOS is observed in intratumoral myeloid cells and is dependent on IFNγ as evidenced using high dimensional profiling [115]. Thus, IFNγ drives the polarization of newly recruited monocytes to become iNOS-positive macrophages. This remodeling by ICT is also correlated with an increase in activated T cells and a decrease in Tregs. Therefore, combining ICT with reactive nitrogen species reducing agents could represent treatment strategies for ICT-resistant cancers.

Growing literature suggests that the induction of senescence in the immune compartment is also a mechanism used by the immune system to regulate the immune response. Human Tregs induce senescence in responder T cells by the regulation of STAT1/STAT3 signaling [116]. Moreover, the autologous infusion of tumor antigen specific CD4^+^ Th1 lymphocyte can promote senescence in pancreatic tumor cells by releasing SASP factors such as IFNγ and TNFα. Such cytokine-induced senescence strictly requires STAT1 and TNFR1 signaling pathways, themselves affected by S-nitrosylation [100].

Since NO is present in the tumor microenvironment associated with senescent cells, it seems important to precisely determine the nitration or S-nitrosylation state of molecules detected in SASP or involved in SASP signaling.

Altogether, several studies put forward arguments to show the immunosuppressive effect of NO. In most cases, these arguments are based on the immunosuppressive effect of endogenous NO produced, in small amounts, by NOSs expressed in immune cells, particularly in MDSCs. On the other hand, NO donors generating high amounts of NO can reduce this immunosuppression. Such effects may be due to the ability of these donors to induce a feedback inhibition of iNOS, a key mechanism of MDSC-mediated immunosuppression. However, few clinical trials have tested the anti-tumor potential of the agonist TLR4, known to induce the production of iNOS and NO [117,118].

## 6. Senolytic Drugs and NO

As discussed in the “senescence and cancer” part, senescence can play an anti- or a pro-tumorigenic role in cancer. In fact, senescent cells correspond to cells that have irreversibly lost their capability to divide but that are very resistant to apoptotic stimuli [119]. We can distinguish six Senescent-Cell Anti-Apoptotic Pathways (SCAPs): BCL-2/BCL-XL, the PI3K/AKT/ceramide metabolic network, MDM2/p53/p21/serpin elements, Ephirins/dependence receptors/tyrosine kinases, the hypoxia inducible factor (HIF-1α) pathway, and the heat shock protein 90 (HSP-90)-dependent pathway [27]. It seems as though the inhibition of these different pathways could induce apoptosis preferentially in senescent cells, which thus could delay age-associated pathologies [119]. Molecules used to induce senescent cell removal are called senolytics. To this group belong dasatinib and quercetin, BCL-2 family inhibitors, Forkhead box O 4 (FOXO4) inhibitors and others such as nicotinamide riboside, danazol, fisetin, piperlongumine and heat shock protein 90 (HSP90) inhibitors [34]. Thus far, many researchers view senolytics as a second wave of adjuvant tumor therapy, following chemotherapy or radiotherapy [120]. At this stage, links also exist between NO and senolytic drugs as described below.

### 6.1. Dasatinib/Quercetin

Dasatinib is a second-generation tyrosine kinase inhibitor that targets several kinases including breakpoint cluster region–protein Abelson (Bcr-Abl), c-Kit, and platelet-derived growth factor receptor A (PDGFRA) and B but also Src kinase family members. It is commonly used in the treatment of imatinib-resistant chronic myeloid leukemia patients [121,122]. Quercetin, instead, is a polyphenol compound that can be found in food like nuts, teas, vegetables and herbs and which is highlighted for its cytotoxic effects in several types of cancer without harming healthy cells [123]. Many modifications can be induced by these two senolytic drugs of NO levels. Indeed, dasatinib can induce an increase in plasma NO in pulmonary artery and smooth muscle cells [124]. Additionally, Hu et al. (2018) recently found that Src inhibition by dasatinib increases iNOS, a pro-inflammatory macrophage marker, in both intestinal and bone marrow-derived macrophages [125]. Conversely, Cruz et al. (2016) reported that dasatinib is able to reduce lung inflammation and fibrosis by promoting the polarization of macrophages from the M1 to M2 phenotype. This effect is due to the ability of dasatinib to reduce iNOS expression specifically in silicotic macrophages [126]. Concerning quercetin, all studies demonstrated that this senolytic drug induces a downregulation of iNOS expression, thus inhibiting nitric oxide production in vivo and in vitro (Table 2) [127,128,129,130,131,132,133].

### 6.2. Bcl-2 Inhibitors

The Bcl2 family is composed of a set of proteins that play an important role in promoting (Bax, Bak, Bid and Bim) but also in inhibiting (Bcl-2, Bcl-xL, and Bcl-W) apoptosis. Thus, the overexpression of anti-apoptotic members such as Bcl-2 or the downregulation or mutation of pro-apoptotic ones may be the cause of acquired resistance to apoptosis and cancer development [136]. Targeting apoptotic inhibitors represents, then, an efficient and promising cancer treatment. Post-translational modifications induced by NO on Bcl-2 have been reported. Indeed, Wright et al. (2016) revealed that Bcl-2 can be S-nitrosylated with NO donor DPTA-NONOate, thus inducing resistance to autophagy initiation in malignantly transformed lung cells [137].

### 6.3. Hsp90 Inhibitors

Hsp90 is a chaperone protein that assists the regulation, the folding and the stabilization/degradation of multiple proteins involved in multiple signaling pathways [138]. Proteins involved in tumor growth (EGFR, HER2, BRAF, Akt, etc.) can also be stabilized by this chaperone protein, leading to cancer development [139]. Hsp90 inhibitors are thus highlighted as anti-cancer drugs. In 1998, Garcia-Cardena et al. reported that Hsp90 can interact with eNOS and enhance its activity to induce NO production [140]. This activation seems to be induced by Hsp90 and Akt [134]. Indeed, it has been demonstrated that the Hsp90-dependent phosphorylation of eNOS at serine 1177 (human eNOS) or 1179 (bovine eNOS) is a key post-translational modification in the initiation of eNOS activation and NO synthesis [141]. The inhibition of Hsp90 by Geldanamycin [134] or taxotere [135] induced a marked reduction in eNOS activity (Table 2). eNOS can also be responsible for post-translational modifications of Hsp90 and further inhibiting eNOS activation. Martínez-Ruiz et al. (2005) were the first to determine that human Hsp90 can be S-nitrosylated at cysteine 597 in endothelial cells, which inhibits its ATPase activity in vitro [142] and in vivo [143].

In order to improve anti-cancer treatments, new combination therapies could be tested to successively induce the therapy-induced senescence (TIS) and the subsequent elimination of senescent cells by the immune system (senotherapy). Indeed, Xue et al. (2007) have shown in a p53 rescue experiment that innate immune cells such as macrophages, neutrophils and NK cells are involved in senescent cell removal [144]. Adaptive immune cells such as the Th1 subclass of CD4^+^ cells are also involved in immune-mediated clearance in a model of OIS in murine hepatocytes [145].

More recently, the combination of senescence-inducing chemotherapy (oxaliplatin or cisplatin) with a TLR4 agonist, the lipid A OM-174, has demonstrated strong anti-tumor efficacy in a model of advanced colon carcinoma [146]. The anti-tumor efficacy of this Lipid A has previously been demonstrated in several models of tumors [147,148,149] and was dependent on cytokine secretion (IFNγ, IL-1β and TNFα), iNOS activation and tumor-associated neutrophil (TAN) reprogramming into anti-tumorigenic N1 [149,150]. Moreover, the safety and tolerance at biologically active concentrations of the lipid A compound have been demonstrated in a phase I clinical trial in patients with cancers of different origins [117]. However, in our animal model with advanced colon carcinoma, lipid A—when used alone (as well as with platinum drugs)—failed to induce tumor regression, while pretreatment with oxaliplatin or cisplatin followed by lipid A injections induced a large regression of colorectal tumors [146]. The anti-tumor effect of this combination is correlated with a sequential induction of (1) cancer cell senescence and the recruitment of pro-tumorigenic TAN (N2), induced by platinum derivative drugs; and (2) apoptosis and N1 TAN recruitment triggered by lipid A.

## 7. Conclusions

In the fight against cancer, the search for new therapeutic combinations remains a major challenge. Senescence and, more particularly, SASP are major targets of these treatments. Indeed, many treatments currently used in anti-cancer therapies induce senescence, which is called therapy-induced senescence (TIS). These treatments include docetaxel, bleomycin, cyclophosphamide, doxorubicin, vincristine, etoposide and oxaliplatin [48,146] but also ionizing radiation [34]. However, senescence and, more particularly, SASP have an early anti-tumorigenic effect whereas in the long term, they will have a pro-tumorigenic role via their autocrine and paracrine actions [151]. In order to avoid the negative side effects of SASP after TIS, a new type of therapy called senotherapy has emerged to either counteract these SASP effects or to specifically eliminate senescent cells. The first strategy consists of counteracting the pro-tumorigenic effects of SASP. This can be achieved with the use of telomerase inhibitors, the therapeutic modulation of the cell cycle, p53 and myc targeting, the use of immunotherapy that targets MDSCs, and the reprogramming of SASP [34]. In this review, we highlighted NO as an innovative approach to modulate SASP effects. Even though NO has been described to induce the senescence of tumor cells [75], it also mainly modulates the expression of SASP factors, as well as other senomorphic agents [34], by regulating numerous signaling pathways such as NF-κB and STAT3 [84,93]. The second therapeutic strategy consists of inducing senescence and subsequently clearing the senescent cells either specifically, by inducing their apoptosis, or by activating the immune system. The use of NO donors as senotherapy seems to be a promising solution since they are described to potentiate the apoptosis induced by death ligands such as FasL, TNFα and TRAIL. In addition, several clinical trials have shown the advantage of using an NO donor such as GTN in combination with TIS agents such as vincristine, cisplatin and radiotherapy in the treatment of non-small cell lung cancer [69,71]. NO, through its immunomodulatory potential, can also be used to eliminate senescent cells. Thus, the combination of TIS agents such as oxaliplatin and cisplatin with an immunomodulatory agent such as lipid A has significant anti-tumor efficacy in an advanced cancer model [146]. Overall, the recent clinical trials that involved either TLR4 agonists [117,118] or NO donors [69,70,71] suggest that these therapies, used alone or in combination with TIS, might be beneficial to treat cancer. These results prompt us to reconsider the commonly accepted pro-tumorigenic effect of NO based on its production by immunosuppressive MDSCs. Further studies will be needed to better understand the pathophysiological conditions that determine whether NO will tip the balance to tumor progression or cancer cure.

## Figures and Tables

**Table 1:** The senescence-associated secretory phenotype (SASP) factors (based on [24,34]).

Interleukins and Other Inflammatory Molecules	IL-1; IL-1β; IL-6; IL-7; IL-8; IL-13; IL-15; TGFβ; GM-CSF; G-CSF; CSF-1; IFN-γ; BLC; MIF
Chemokines and Growth Factors/Regulators	CXCL1; CXCL2; CXCL5; CXCL12; CCL2; CCL5; CCL8; CCL13; MIP-1α; MIP-3α; HCC-4; eotaxin/eotaxin-3; TECK; ENA-78; Amphiregulin; epiregulin; heregulin; EGF; bFGF; HGF; KGF (FGF7); VEGF; angiogenin; SCF
Receptors and Ligands	ICAM-1/3; OPG; TNFα; sTNFRI; sTNFRII; TRAIL-R3; Fas; uPAR; SGP130; EGF-R
Proteases and Extracellular Matrix Proteins	MMP-1/3/10/12/13/14; TIMP-1/2; PAI-1/2; tPA; uPA; cathepsin B
Non-Protein Molecules and Insoluble Factors	Nitric oxide; ROS; PGE2; fibronectin; collagens; laminin

**Table 2:** Senolytic-induced nitric oxide (NO) modulations.

Senolytic Drugs	NO Changes	Model	References
Dasatinib	Increased NO	Pulmonary artery endothelial cells and smooth muscle cells	[124]
	Increase iNOS expresion	Intestinal and bone morrow-derived macrophages	[125]
	Decrease iNOS expresion	Silicotic macrophages	[126]
Quercetin	Inhibition of iNOS		[127]
	Inhibition of mRNA iNOS	Human hepatocyte-derived cell line	[128]
	Inhibition of NO production and iNOS expression	Livers of CCl4-treated mice	[129]
		In vitro (rat hepatocyte)	[130]
		In vitro and in vivo (RAW 264.7 macrophages, primary peritoneal macrophages and Balb/c mice)	[131]
		Chronic cadmium nephrotoxicity in rats	[132]
		Lung adenocarcinoma cell lines	[133]
Hsp90 inhibitor	Reduction of NO production	Endothelial cells	[134]
	Blocked VEGF-induced increase in eNOS activity	Endothelial cells	[135]
